# Di­aqua­bis­(3-chloro­benzoato-κ*O*)bis­(nicotinamide-κ*N* ^1^)cobalt(II)

**DOI:** 10.1107/S160053681301458X

**Published:** 2013-05-31

**Authors:** Nihat Bozkurt, Nefise Dilek, Nagihan Çaylak Delibaş, Hacali Necefoğlu, Tuncer Hökelek

**Affiliations:** a Department of Chemistry, Kafkas University, 36100 Kars, Turkey; b Aksaray University, Department of Physics, 68100, Aksaray, Turkey; c Department of Physics, Sakarya University, 54187 Esentepe, Sakarya, Turkey; d Department of Physics, Hacettepe University, 06800 Beytepe, Ankara, Turkey

## Abstract

In the title complex, [Co(C_7_H_4_ClO_2_)_2_(C_6_H_6_N_2_O)_2_(H_2_O)_2_], the Co^II^ atom is located on an inversion center and is coordinated by two 3-chloro­benzoate (CB) anions, two nicotinamide (NA) ligands and two water mol­ecules. The four O atoms in the equatorial plane form a slightly distorted square-planar arrangement, while the slightly distorted octa­hedral coordination is completed by the two N atoms of the NA ligands in the axial positions. The dihedral angle between the carboxyl­ate group and the adjacent benzene ring is 9.14 (9)°, while the pyridine and benzene rings are oriented at a dihedral angle of 82.18 (8)°. In the crystal, N—H⋯O and O—H⋯O hydrogen bonds link the mol­ecules into a two-dimensional network lying parallel to (101). π–π stacking between parallel pyridine rings of adjacent mol­ecules [centroid–centroid distance = 3.7765 (8) Å] further stabilizes the crystal structure.

## Related literature

For literature on niacin, see: Krishnamachari (1974 ▶). For information on the nicotinic acid derivative *N*,*N*-di­ethyl­nicotinamide, see: Bigoli *et al.* (1972 ▶). For related structures, see: Aydın *et al.* (2012 ▶); Hökelek *et al.* (1996 ▶, 2009*a* ▶,*b* ▶); Hökelek & Necefoğlu (1998 ▶, 2007 ▶); Necefoğlu *et al.* (2011*a* ▶,*b* ▶); Sertçelik *et al.* (2012*a* ▶,*b* ▶,*c* ▶). For bond-length data, see: Allen *et al.* (1987 ▶).
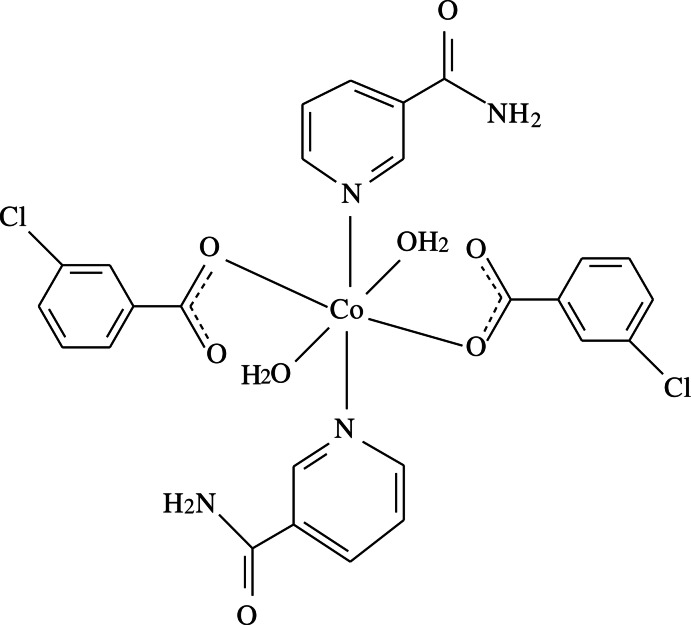


## Experimental

### 

#### Crystal data

[Co(C_7_H_4_ClO_2_)_2_(C_6_H_6_N_2_O)_2_(H_2_O)_2_]
*M* *_r_* = 650.32
Monoclinic, 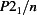

*a* = 11.5181 (3) Å
*b* = 8.8191 (2) Å
*c* = 13.5089 (3) Å
β = 90.546 (2)°
*V* = 1372.16 (6) Å^3^
*Z* = 2
Mo *K*α radiation
μ = 0.88 mm^−1^
*T* = 294 K
0.35 × 0.22 × 0.18 mm

#### Data collection

Bruker APEXII CCD area-detector diffractometer
Absorption correction: multi-scan (*SADABS*; Bruker, 2012 ▶) *T*_min_ = 0.793, *T*_max_ = 0.854
18960 measured reflections
2797 independent reflections
2667 reflections with *I* > 2σ(*I*)
*R*_int_ = 0.027

#### Refinement

*R*[*F* ^2^ > 2σ(*F* ^2^)] = 0.027
*wR*(*F* ^2^) = 0.074
*S* = 1.11
2797 reflections
204 parameters
52 restraints
H atoms treated by a mixture of independent and constrained refinement
Δρ_max_ = 0.32 e Å^−3^
Δρ_min_ = −0.33 e Å^−3^

### 

Data collection: *APEX2* (Bruker, 2012 ▶); cell refinement: *SAINT* (Bruker, 2012 ▶); data reduction: *SAINT*; program(s) used to solve structure: *SHELXS97* (Sheldrick, 2008 ▶); program(s) used to refine structure: *SHELXL97* (Sheldrick, 2008 ▶); molecular graphics: *ORTEP-3 for Windows* (Farrugia, 2012 ▶); software used to prepare material for publication: *WinGX* (Farrugia, 2012 ▶) and *PLATON* (Spek, 2009 ▶).

## Supplementary Material

Crystal structure: contains datablock(s) I, global. DOI: 10.1107/S160053681301458X/su2606sup1.cif

Structure factors: contains datablock(s) I. DOI: 10.1107/S160053681301458X/su2606Isup2.hkl

Additional supplementary materials:  crystallographic information; 3D view; checkCIF report

## Figures and Tables

**Table 1:** Hydrogen-bond geometry (Å, °)

*D*—H⋯*A*	*D*—H	H⋯*A*	*D*⋯*A*	*D*—H⋯*A*
N2—H21⋯O3^i^	0.84 (3)	2.24 (3)	2.876 (2)	133 (2)
N2—H22⋯O4^ii^	0.87 (2)	2.27 (2)	3.012 (2)	143 (2)
O4—H41⋯O1^iii^	0.92 (2)	1.68 (2)	2.5822 (16)	164 (2)
O4—H42⋯O3^iv^	0.83 (3)	1.98 (3)	2.7892 (15)	166 (2)

Symmetry codes: (i) 
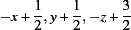
; (ii) 
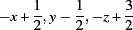
; (iii) 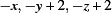; (iv) 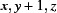.

**Fig. 1.**
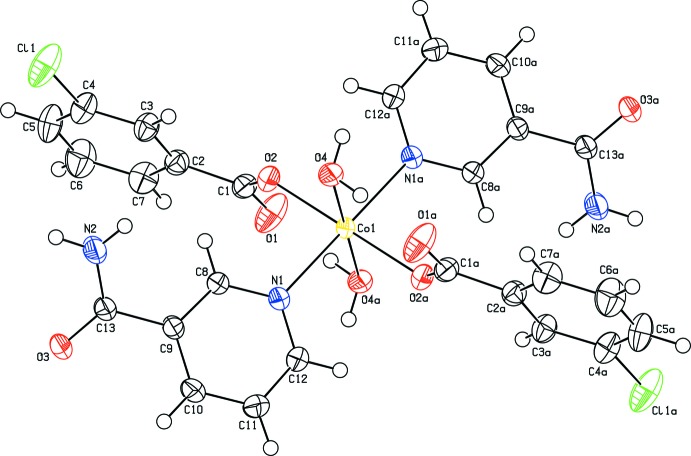
The molecular structure of the title molecule, with atom labelling. Displacement ellipsoids are drawn at the 50% probability level [symmetry code: (a) - x, - y, - z].

## supplementary crystallographic information

### Comment

As a part of our ongoing investigations of transition metal complexes of nicotinamide (NA), one form of niacin (Krishnamachari, 1974), and/or the nicotinic acid derivative *N*,*N*-diethylnicotinamide (DENA), an important respiratory stimulant (Bigoli *et al.*, 1972), the title compound was synthesized and its crystal structure is reported herein.

In the title mononuclear complex, Co^II^ atom is located on an inversion center and is coordinated by two 3-chlorobenzoate (CB) anions, two nicotinamide (NA) ligands and two water molecules, all ligands coordinating in a monodentate manner (Fig. 1). The crystal structures of similar complexes of Cu^II^, Co^II^, Ni^II^, Mn^II^ and Zn^II^ ions, [Cu(C_7_H_5_O_2_)_2_(C_10_H_14_N_2_O)_2_] (Hökelek *et al.*, 1996), [Cu(C_7_H_4_BrO_2_)_2_(C_6_H_6_N_2_O)_2_(H_2_O)_2_] (Necefoğlu *et al.*, 2011*a*), [Co(C_6_H_6_N_2_O)_2_(C_7_H_4_NO_4_)_2_(H_2_O)_2_] (Hökelek & Necefoğlu, 1998), [Co(C_9_H_9_O_2_)_2_(C_10_H_14_N_2_O)_2_(H_2_O)_2_] (Necefoğlu *et al.*, 2011*b*), [Co(C_7_H_4_IO_2_)_2_(C_6_H_6_N_2_O)_2_(H_2_O)_2_] (Aydın *et al.*, 2012), [Co(C_8_H_5_O_3_)_2_(C_6_H_6_N_2_O)_2_(H_2_O)_2_] (Sertçelik *et al.*, 2012*a*), [Ni(C_7_H_4_ClO_2_)_2_(C_6_H_6_N_2_O)_2_(H_2_O)_2_] (Hökelek *et al.*, 2009*a*), [Ni(C_5_H_5_O_3_)_2_(C_6_H_6_N_2_O)_2_(H_2_O)_2_] (Sertçelik *et al.*, 2012*b*), [Mn(C_9_H_10_NO_2_)_2_(H_2_O)_4_].2H_2_O (Hökelek & Necefoğlu, 2007), [Zn(C_7_H_4_BrO_2_)_2_(C_6_H_6_N_2_O)_2_(H_2_O)_2_] (Hökelek *et al.*, 2009*b*) and [Zn(C_8_H_5_O_3_)_2_(C_10_H_14_N_2_O)_2_(H_2_O)_2_] (Sertçelik *et al.*, 2012*c*) have been reported. In the copper(II) complex mentioned above the two benzoate ions coordinate to the Cu^II^ atom as bidentate ligands, while in the other structures all the ligands coordinate in a monodentate manner.

In the title complex, Fig. 1, the four symmetry related O atoms (O2, O2a, O4 and O4a) [symmetry code: (a) - *x*, - *y*, - *z*] in the equatorial plane around the Co^II^ ion form a slightly distorted square-planar arrangement, while the slightly distorted octahedral coordination is completed by the two symmetry related N atoms of the NA ligands (N1 and N1a) in the axial positions. The near equalities of the C1—O1 [1.2435 (19) Å] and C1—O2 [1.2677 (18) Å] bonds in the carboxylate group indicate delocalized bonding arrangement, rather than localized single and double bonds. The Co—O bond lengths are 2.0592 (10) Å (for benzoate oxygens) and 2.1385 (10) Å (for water oxygens), and the Co—N bond length is 2.1641 (11) Å, close to standard values (Allen *et al.*, 1987). The Co atom is displaced out of the mean-plane of the carboxylate group (O1/C1/O2) by -0.5077 (1) Å. The dihedral angle between the planar carboxylate group and the adjacent benzene ring (C2—C7) is 9.14 (9)°. The benzene (C2—C7) and the pyridine (N1/C8—C12) rings are oriented at a dihedral angle of 82.18 (8)°.

In the crystal, N—H···O and O—H···O hydrogen bonds (Table 1) link the molecules into a two-dimensional network lying parallel to (101). π···π stacking between the pyridine rings, Cg···Cg^i^ [symmetry code: (i) -x, -y+1, -z+2, where Cg is the centroid of ring N1/C8—C12] further stabilizes the crystal structure, with a centroid-centroid distance of 3.7765 (8) Å.

### Experimental

The title compound was prepared by the reaction of CoSO_4_.H_2_O (0.865 g, 5 mmol) in H_2_O (25 ml) and nicotinamide (1.22 g, 50 mmol) in H_2_O (100 ml) with sodium 3-chlorobenzoate (1.79 g, 10 mmol) in H_2_O (100 ml) at room temperature. The mixture was filtered and set aside to crystallize at ambient temperature for several days, giving pink single crystals.

### Refinement

Atoms H21 and H22 (for NH_2_) and H41 and H42 (for H_2_O) were located in a difference Fourier map and were refined freely. The C-bound H-atoms were positioned geometrically with C—H = 0.93 Å for aromatic H-atoms, and constrained to ride on their parent atoms, with *U*_iso_(H) = 1.2 × *U*_eq_(C).

### Crystal data

**Table:**

[Co(C_7_H_4_ClO_2_)_2_(C_6_H_6_N_2_O)_2_(H_2_O)_2_]	*F*(000) = 666
*M**_r_* = 650.32	*D*_x_ = 1.574 Mg m^−^^3^
Monoclinic, *P*2_1_/*n*	Mo *K*α radiation, λ = 0.71073 Å
Hall symbol: -P 2yn	Cell parameters from 4857 reflections
*a* = 11.5181 (3) Å	θ = 2.3–24.4°
*b* = 8.8191 (2) Å	µ = 0.88 mm^−^^1^
*c* = 13.5089 (3) Å	*T* = 294 K
β = 90.546 (2)°	Block, pink
*V* = 1372.16 (6) Å^3^	0.35 × 0.22 × 0.18 mm
*Z* = 2	

### Data collection

**Table:**

Bruker APEXII CCD area-detector diffractometer	2797 independent reflections
Radiation source: fine-focus sealed tube	2667 reflections with *I* > 2σ(*I*)
Graphite monochromator	*R*_int_ = 0.027
φ and ω scans	θ_max_ = 26.4°, θ_min_ = 2.3°
Absorption correction: multi-scan (*SADABS*; Bruker, 2012)	*h* = −14→14
*T*_min_ = 0.793, *T*_max_ = 0.854	*k* = −11→10
18960 measured reflections	*l* = −16→16

### Refinement

**Table:**

Refinement on *F*^2^	Secondary atom site location: difference Fourier map
Least-squares matrix: full	Hydrogen site location: inferred from neighbouring sites
*R*[*F*^2^ > 2σ(*F*^2^)] = 0.027	H atoms treated by a mixture of independent and constrained refinement
*wR*(*F*^2^) = 0.074	*w* = 1/[σ^2^(*F*_o_^2^) + (0.0393*P*)^2^ + 0.5258*P*] where *P* = (*F*_o_^2^ + 2*F*_c_^2^)/3
*S* = 1.11	(Δ/σ)_max_ = 0.001
2797 reflections	Δρ_max_ = 0.32 e Å^−^^3^
204 parameters	Δρ_min_ = −0.33 e Å^−^^3^
52 restraints	Extinction correction: *SHELXL97* (Sheldrick, 2008), Fc^*^=kFc[1+0.001xFc^2^λ^3^/sin(2θ)]^-1/4^
Primary atom site location: structure-invariant direct methods	Extinction coefficient: 0.0334 (17)

### Special details

**Table:**

Geometry. Bond distances, angles etc. have been calculated using the rounded fractional coordinates. All su's are estimated from the variances of the (full) variance-covariance matrix. The cell esds are taken into account in the estimation of distances, angles and torsion angles
Refinement. Refinement of F^2^ against ALL reflections. The weighted R-factor wR and goodness of fit S are based on F^2^, conventional R-factors R are based on F, with F set to zero for negative F^2^. The threshold expression of F^2^ > 2sigma(F^2^) is used only for calculating R-factors(gt) etc. and is not relevant to the choice of reflections for refinement. R-factors based on F^2^ are statistically about twice as large as those based on F, and R- factors based on ALL data will be even larger.

### Fractional atomic coordinates and isotropic or equivalent isotropic displacement parameters (Å^2^)

**Table:**

	*x*	*y*	*z*	*U*_iso_*/*U*_eq_	
Co1	0.00000	1.00000	1.00000	0.0221 (1)	
Cl1	0.57400 (4)	0.77742 (8)	0.87425 (3)	0.0604 (2)	
O1	0.17264 (10)	0.87980 (18)	1.18080 (9)	0.0518 (4)	
O2	0.17291 (8)	0.94984 (12)	1.02212 (8)	0.0299 (3)	
O3	0.16810 (10)	0.34420 (12)	0.81282 (10)	0.0415 (4)	
O4	0.04558 (10)	1.07995 (12)	0.85606 (8)	0.0298 (3)	
N1	−0.02319 (10)	0.77771 (13)	0.93492 (9)	0.0258 (3)	
N2	0.23334 (16)	0.56716 (18)	0.76095 (16)	0.0592 (6)	
C1	0.21833 (12)	0.88839 (17)	1.09796 (11)	0.0293 (4)	
C2	0.33518 (12)	0.81535 (17)	1.08465 (11)	0.0286 (4)	
C3	0.39495 (13)	0.83285 (18)	0.99657 (11)	0.0316 (4)	
C4	0.49988 (13)	0.7599 (2)	0.98487 (12)	0.0368 (5)	
C5	0.54679 (15)	0.6700 (2)	1.05879 (14)	0.0464 (6)	
C6	0.48691 (17)	0.6533 (2)	1.14586 (14)	0.0496 (6)	
C7	0.38168 (15)	0.7255 (2)	1.15907 (12)	0.0388 (5)	
C8	0.06524 (12)	0.70512 (15)	0.89207 (11)	0.0268 (4)	
C9	0.05526 (12)	0.56102 (15)	0.85229 (10)	0.0258 (4)	
C10	−0.05151 (14)	0.48869 (16)	0.85705 (12)	0.0314 (4)	
C11	−0.14353 (13)	0.56408 (18)	0.89921 (13)	0.0356 (5)	
C12	−0.12591 (12)	0.70790 (16)	0.93721 (11)	0.0303 (4)	
C13	0.15664 (14)	0.48213 (16)	0.80673 (12)	0.0304 (4)	
H3	0.36460	0.89300	0.94610	0.0380*	
H5	0.61770	0.62160	1.04990	0.0560*	
H6	0.51750	0.59290	1.19620	0.0590*	
H7	0.34200	0.71360	1.21820	0.0470*	
H8	0.13670	0.75380	0.88890	0.0320*	
H10	−0.06090	0.39100	0.83220	0.0380*	
H11	−0.21640	0.51890	0.90200	0.0430*	
H12	−0.18830	0.75810	0.96560	0.0360*	
H21	0.221 (2)	0.659 (3)	0.7492 (19)	0.066 (7)*	
H22	0.293 (2)	0.526 (3)	0.7323 (19)	0.063 (7)*	
H41	−0.028 (2)	1.098 (3)	0.8311 (18)	0.069 (7)*	
H42	0.079 (2)	1.163 (3)	0.8530 (17)	0.055 (6)*	

### Atomic displacement parameters (Å^2^)

**Table:**

	*U*^11^	*U*^22^	*U*^33^	*U*^12^	*U*^13^	*U*^23^
Co1	0.0201 (2)	0.0183 (2)	0.0280 (2)	0.0011 (1)	0.0066 (1)	−0.0026 (1)
Cl1	0.0389 (2)	0.1013 (4)	0.0412 (3)	0.0171 (2)	0.0140 (2)	0.0029 (2)
O1	0.0339 (6)	0.0875 (10)	0.0342 (6)	0.0108 (6)	0.0090 (5)	0.0038 (6)
O2	0.0230 (5)	0.0306 (5)	0.0362 (5)	0.0039 (4)	0.0044 (4)	0.0007 (4)
O3	0.0403 (6)	0.0194 (5)	0.0651 (8)	0.0024 (4)	0.0211 (5)	−0.0007 (5)
O4	0.0297 (5)	0.0267 (6)	0.0332 (5)	−0.0023 (4)	0.0098 (4)	−0.0001 (4)
N1	0.0256 (6)	0.0205 (5)	0.0313 (6)	0.0005 (4)	0.0049 (5)	−0.0027 (4)
N2	0.0569 (10)	0.0231 (7)	0.0985 (14)	0.0036 (7)	0.0507 (10)	0.0049 (8)
C1	0.0247 (7)	0.0308 (7)	0.0324 (7)	−0.0020 (5)	0.0034 (5)	−0.0042 (6)
C2	0.0261 (7)	0.0297 (7)	0.0301 (7)	0.0001 (6)	−0.0001 (5)	−0.0025 (6)
C3	0.0273 (7)	0.0369 (8)	0.0307 (7)	0.0051 (6)	0.0000 (6)	0.0031 (6)
C4	0.0286 (7)	0.0489 (10)	0.0331 (8)	0.0054 (6)	0.0038 (6)	−0.0028 (7)
C5	0.0344 (8)	0.0564 (11)	0.0482 (10)	0.0200 (8)	−0.0027 (7)	0.0004 (8)
C6	0.0479 (10)	0.0572 (11)	0.0435 (10)	0.0165 (9)	−0.0075 (8)	0.0127 (8)
C7	0.0401 (9)	0.0459 (9)	0.0303 (8)	0.0038 (7)	0.0005 (6)	0.0045 (7)
C8	0.0251 (6)	0.0214 (6)	0.0339 (7)	−0.0006 (5)	0.0068 (5)	−0.0010 (5)
C9	0.0292 (7)	0.0197 (6)	0.0287 (7)	0.0017 (5)	0.0063 (5)	0.0004 (5)
C10	0.0336 (8)	0.0220 (7)	0.0388 (8)	−0.0030 (5)	0.0051 (6)	−0.0062 (5)
C11	0.0270 (7)	0.0312 (8)	0.0486 (9)	−0.0058 (6)	0.0062 (6)	−0.0072 (7)
C12	0.0249 (7)	0.0276 (7)	0.0384 (8)	0.0010 (5)	0.0073 (6)	−0.0032 (6)
C13	0.0320 (8)	0.0211 (7)	0.0382 (8)	0.0000 (5)	0.0112 (6)	−0.0025 (6)

### Geometric parameters (Å, º)

**Table:**

Co1—O2	2.0592 (9)	C2—C3	1.389 (2)
Co1—O4	2.1385 (11)	C3—C4	1.380 (2)
Co1—N1	2.1640 (12)	C4—C5	1.381 (2)
Co1—O2^i^	2.0592 (9)	C5—C6	1.377 (3)
Co1—O4^i^	2.1385 (11)	C6—C7	1.382 (3)
Co1—N1^i^	2.1640 (12)	C8—C9	1.3841 (19)
Cl1—C4	1.7350 (16)	C9—C13	1.497 (2)
O1—C1	1.2435 (19)	C9—C10	1.387 (2)
O2—C1	1.2676 (18)	C10—C11	1.379 (2)
O3—C13	1.2262 (18)	C11—C12	1.383 (2)
O4—H41	0.92 (2)	C3—H3	0.9300
O4—H42	0.83 (3)	C5—H5	0.9300
N1—C12	1.3344 (18)	C6—H6	0.9300
N1—C8	1.3392 (18)	C7—H7	0.9300
N2—C13	1.317 (2)	C8—H8	0.9300
N2—H21	0.84 (3)	C10—H10	0.9300
N2—H22	0.87 (2)	C11—H11	0.9300
C1—C2	1.504 (2)	C12—H12	0.9300
C2—C7	1.384 (2)		
			
O2—Co1—O4	87.55 (4)	Cl1—C4—C3	119.73 (12)
O2—Co1—N1	88.84 (4)	C3—C4—C5	121.47 (15)
O2—Co1—O2^i^	180.00	Cl1—C4—C5	118.79 (13)
O2—Co1—O4^i^	92.45 (4)	C4—C5—C6	118.90 (16)
O2—Co1—N1^i^	91.16 (4)	C5—C6—C7	120.53 (17)
O4—Co1—N1	87.69 (4)	C2—C7—C6	120.25 (15)
O2^i^—Co1—O4	92.45 (4)	N1—C8—C9	123.09 (13)
O4—Co1—O4^i^	180.00	C8—C9—C13	121.62 (13)
O4—Co1—N1^i^	92.31 (4)	C8—C9—C10	118.33 (13)
O2^i^—Co1—N1	91.16 (4)	C10—C9—C13	120.05 (12)
O4^i^—Co1—N1	92.31 (4)	C9—C10—C11	118.86 (13)
N1—Co1—N1^i^	180.00	C10—C11—C12	119.01 (14)
O2^i^—Co1—O4^i^	87.55 (4)	N1—C12—C11	122.80 (13)
O2^i^—Co1—N1^i^	88.84 (4)	N2—C13—C9	117.24 (13)
O4^i^—Co1—N1^i^	87.69 (4)	O3—C13—N2	121.59 (16)
Co1—O2—C1	126.89 (9)	O3—C13—C9	121.16 (14)
H41—O4—H42	105 (2)	C2—C3—H3	120.00
Co1—O4—H41	99.0 (15)	C4—C3—H3	120.00
Co1—O4—H42	117.1 (16)	C4—C5—H5	121.00
Co1—N1—C8	121.14 (9)	C6—C5—H5	121.00
Co1—N1—C12	120.97 (9)	C5—C6—H6	120.00
C8—N1—C12	117.88 (12)	C7—C6—H6	120.00
H21—N2—H22	117 (2)	C2—C7—H7	120.00
C13—N2—H21	121.8 (16)	C6—C7—H7	120.00
C13—N2—H22	120.5 (17)	N1—C8—H8	118.00
O1—C1—O2	125.34 (14)	C9—C8—H8	118.00
O1—C1—C2	117.92 (13)	C9—C10—H10	121.00
O2—C1—C2	116.70 (13)	C11—C10—H10	121.00
C1—C2—C3	120.41 (13)	C10—C11—H11	121.00
C1—C2—C7	119.92 (13)	C12—C11—H11	120.00
C3—C2—C7	119.63 (14)	N1—C12—H12	119.00
C2—C3—C4	119.23 (14)	C11—C12—H12	119.00
			
O4—Co1—O2—C1	−177.85 (12)	O2—C1—C2—C7	−169.65 (14)
N1—Co1—O2—C1	−90.11 (12)	C1—C2—C3—C4	−177.53 (14)
O4^i^—Co1—O2—C1	2.15 (12)	C7—C2—C3—C4	0.1 (2)
N1^i^—Co1—O2—C1	89.89 (12)	C1—C2—C7—C6	177.50 (15)
O2—Co1—N1—C8	−26.16 (11)	C3—C2—C7—C6	−0.1 (2)
O2—Co1—N1—C12	153.10 (11)	C2—C3—C4—Cl1	178.62 (12)
O4—Co1—N1—C8	61.44 (11)	C2—C3—C4—C5	0.0 (2)
O4—Co1—N1—C12	−119.31 (11)	Cl1—C4—C5—C6	−178.62 (14)
O2^i^—Co1—N1—C8	153.84 (11)	C3—C4—C5—C6	0.1 (3)
O2^i^—Co1—N1—C12	−26.90 (11)	C4—C5—C6—C7	−0.1 (3)
O4^i^—Co1—N1—C8	−118.56 (11)	C5—C6—C7—C2	0.1 (3)
O4^i^—Co1—N1—C12	60.69 (11)	N1—C8—C9—C10	0.1 (2)
Co1—O2—C1—O1	−18.0 (2)	N1—C8—C9—C13	−178.77 (13)
Co1—O2—C1—C2	159.69 (10)	C8—C9—C10—C11	1.3 (2)
Co1—N1—C8—C9	177.93 (11)	C13—C9—C10—C11	−179.86 (14)
C12—N1—C8—C9	−1.4 (2)	C8—C9—C13—O3	146.12 (16)
Co1—N1—C12—C11	−177.98 (12)	C8—C9—C13—N2	−33.2 (2)
C8—N1—C12—C11	1.3 (2)	C10—C9—C13—O3	−32.7 (2)
O1—C1—C2—C3	−174.24 (15)	C10—C9—C13—N2	148.03 (17)
O1—C1—C2—C7	8.2 (2)	C9—C10—C11—C12	−1.3 (2)
O2—C1—C2—C3	7.9 (2)	C10—C11—C12—N1	0.0 (2)

Symmetry code: (i) −*x*, −*y*+2, −*z*+2.

### Hydrogen-bond geometry (Å, º)

**Table:**

*D*—H···*A*	*D*—H	H···*A*	*D*···*A*	*D*—H···*A*
N2—H21···O3^ii^	0.84 (3)	2.24 (3)	2.876 (2)	133 (2)
N2—H22···O4^iii^	0.87 (2)	2.27 (2)	3.012 (2)	143 (2)
O4—H41···O1^i^	0.92 (2)	1.68 (2)	2.5822 (16)	164 (2)
O4—H42···O3^iv^	0.83 (3)	1.98 (3)	2.7892 (15)	166 (2)

Symmetry codes: (i) −*x*, −*y*+2, −*z*+2; (ii) −*x*+1/2, *y*+1/2, −*z*+3/2; (iii) −*x*+1/2, *y*−1/2, −*z*+3/2; (iv) *x*, *y*+1, *z*.
